# Development of Multiwalled Carbon Nanotube-Reinforced Biodegradable Polylactic Acid/Polybutylene Succinate Blend Membrane

**DOI:** 10.3390/membranes11100760

**Published:** 2021-09-30

**Authors:** Badar M. AlruwailI, Usman Saeed, Iqbal Ahmad, Hamad Al-Turaif, Hani Aboalkhair, Abdulmohsen O. AlsaiarI

**Affiliations:** 1 Chemical & Materials Engineering Department, Faculty of Engineering, King Abdulaziz University, Jeddah 21442, Saudi Arabia; balruwaili0002@stu.kau.edu.sa (B.M.A.); halturaif@hotmail.com (H.A.-T.); 2 Center of Excellence in Desalination Technology, King Abdulaziz University, Jeddah 21442, Saudi Arabia; irajboot@kau.edu.sa (I.A.); haboalkhair@kau.edu.sa (H.A.); aoalsaiari@kau.edu.sa (A.O.A.); 3 Mechanical Engineering Department, King Abdulaziz University, Jeddah 21442, Saudi Arabia

**Keywords:** polylactic acid (PLA), polybutylene succinate (PBS), multiwalled carbon nanotubes (MWCNT), membrane

## Abstract

Currently, gas separation (GS) membranes are produced from petrochemical-based polymers, but their lifespan is severely impacting the environment. Therefore, there has recently been growing interest in developing ecofriendly biodegradable polymer-based GS membranes. This study developed a polylactic acid (PLA)/polybutylene succinate (PBS) blend composite membrane for GS using the dry/wet phase inversion technique. The influence of the multiwalled carbon nanotube (MWCNT) concentration in the PLA/PBS blend was studied by investigating tensile properties, porosity, percentage crystallinity, contact angle, and gas permeance.The obtained results demonstrate that the addition of MWCNT enhances the tensile strength, porosity, and percentage crystallinity, whereas it decreases the contact angle. The pure gas permeation was investigated at pressures of 2–4 bar at 25 °C. The gas permeation study revealed that the PLA/PBS blend with 0.5% wt. MWCNT enhanced the gas permeance and selectivity at 4 bar. The gas permeance acquired at 25 °C and 4 bar for PLA/PBS reinforced with MWCNT was highest in hydrogen followed by carbon dioxide, argon, and nitrogen. Additionally, a study of the membrane morphology illustrated the uniform dispersion of MWCNT in the PLA/PBS blend. The investigation concluded that membranes containing MWCNT are capable of separating gases at the molecular level, thereby reducing energy consumption.

## 1. Introduction

Investigations have been conducted to identify and improve appropriate polymeric materials for gas separation. Many studies focused on the relationship between polymeric structure and gas transport [1,2,3,4,5,6]. There are still challenges associated with the development of polymeric membranes related to selectivity and gas permeance [7]. Most studies focused on investigating the influence of polymeric structure on gas separation to find a membrane that functions effectively for both permeance and selectivity. The investigations illustrated that free volume, packing density, and chain mobility in the polymer structure affect the quality of permeance for any polymeric membrane. The technique most effective is when a rigid filler with size equal to the macromolecules constituting the selective polymer membrane is used. The introduction of fillers enhances gas permeability by controlling chain packing and free volume in the polymer structure. Furthermore, other investigations used polymer blend membranes (PBMs), a combination of organic polymers and inorganic nanofillers, to improve selectivity and permeance for gas separation [8,9]. The studies found that the nature of inorganic fillers and organic polymers greatly affects membrane properties such as membrane morphology and gas separation capability [10].

Previous research also used inorganic nanofillers in the form of carbon nanotubes within the organic polymer, which improved gas permeability compared to neat polymer [11]. Kim et al. demonstrated the influence of carbon nanotubes when used in polyimide siloxane polymer for membrane development to increase the permeability of N_2_, CH_4_, and O_2_ [12]. Cong et al. [13] developed a brominated poly(2,6-diphenyl-1,4-phenylene oxide) polymer with carbon nanotubes to demonstrate that the permeability improved with no effect on the selectivity, even with the addition of a low concentration of nanotubes. Weng et al. [14] developed a MWCNT/PBNPI membrane, which improved selectivity and permeance at a high concentration of 5 wt.% MWCNT for CH_4_, CO_2_, and H_2_. These studies showed an essential correlation between polymeric structure and nanotubes, improving gas diffusion, permeance, and selectivity. Furthermore, the introduction of nanotubes affects the polymer chain packing as it introduces accessible volume voids, thereby creating an overall structure suitable for gas separation. The polymeric materials are used in limited quantity as there are still improvements required in polymeric systems to make them commercially viable and cost-effective for membrane manufacturing [15,16,17]. The challenges when developing an efficient membrane have been shown to be high selectivity, permeance, and mechanical resistance [18,19,20]. The PLA lactide monomer produced from lactic acid is nontoxic with appropriate thermal plasticity, mechanical properties, and biocompatibility. The characteristics of PLA limiting its use are its ring-opening polymerization and brittleness. Therefore, to overcome the limitation of PLA in terms of tensile properties, it can be blended into another polymer that can improve the weaknesses found in PLA to develop a cost-effective final product that is helpful in gas separation applications. PBS, produced via the polycondensation of 1,4-butanediol with succinic acid, was selected to be blended with PLA [21]. Biodegradable PBS has qualities that can overcome the deficiencies related to PLA, such as suitable flexibility, impact strength, and thermal and chemical characteristics [22,23].

The study presents biodegradable membrane development by utilizing an inorganic MWCNT-reinforced PLA/PBS blend. The characteristics of the membrane investigated were crystallinity, porosity, hydrophilicity, tensile strength, and morphology. Moreover, the selectivity and permeance of the membrane were analyzed by utilizing Ar, H_2_, CO_2_, and N_2_ gases at 2–4 bar and 25 °C.

## 2. Materials and Methods

### 2.1. Preparation of Membrane

Nature Works, USA, supplied polylactic acid (PLA-2003D), and Showa Denko K.K. Japan provided polybutylene succinate (PBS-3001MD). Hydroxylated functionalized multiwalled carbon nanotubes (MWCNTs) with >96% purity and 8–18 nm diameter were purchased from NanografiNano Technology USA. Merck, Darmstadt, Germany supplied the chloroform (CHL), used as the organic solvent, in addition to the industrial-grade Ethanol (ETOH) of purity 97% ± 1%, as a coagulant medium for the phase inversion process, and deionized water (DIW), used to wash and anneal (60 °C) the membranes.

A 50:50 ratio was used to blend PLA–PBS. The MWCNT was added in varying concentrations from 0.1% to 0.5% into the PLA/PBS blend. Before dissolving the polymers and MWCNT, the PLA–PBS at a 50:50 ratio was dried in a vacuum oven at 60 °C for 24 h, and the dope solution was prepared with 18 wt.% PLA–PBS. To avoid agglomeration, the MWCNT was first incorporated in chloroform and stirred continuously at 40 °C. Later, the PLA/PBS blend was gradually added and mixed continually at 65 °C for 4 h at 500 rpm to develop a homogeneous solution. Before casting, to avoid any microbubbles, the prepared solution was degassed by utilizing an ultrasonic bath. The pneumatically controlled casting machine was utilized for the casting process. The MWCNT/PLA/PBS dope solution was cast on a glass plate with a knife gap of 300 μm at a suitable casting shear. The casted membrane was immersed in a coagulation bath of ethanol at a temperature of 25 °C. After DIW washing and annealing, the fabricated MWCNT/PLA/PBS membranes were dried in air for 24 h at room temperature.

### 2.2. Characterization

A differential scanning calorimeter (DSC 200 F3) (Netzsch, Selb, Germany) was used to analyze theMWCNT/PLA/PBS membrane. First, 10–15 mg samples were heated from 20 °C to 200 °C at a heating rate of 2 °C/min and cooled to room temperature at the same rate. The thermograms were utilized to study the melting enthalpy, melting temperature, and glass transition temperature. Equation (1) was used to calculate the degree of crystallinity (% *X_C_*),

(1) $$X_{c}\;\%=\frac{\Delta H_{m}}{w.\Delta H_{m}^{o}}\times 100,$$

where Δ*Hm* is the experimental melting enthalpy (J/g) for the polymer blend, w is the weight fraction of PLA or PBS, and Δ*H*^0^*m* is the melting enthalpy of 100% crystalline PLA (93.7 J/g) and PBS (110.3 J/g) [23].

FTIR analysis of PLA/PBS and PLA/PBS/ MWCNT membranes was performed at ambient temperature using a Thermo Scientific Nicolet 6700 ATR-FTIR (Thermo Fisher Scientific, Waltham, MA, USA). The spectra were characterized in the wavelength range of 400–4000 cm^−1^ with a step size of 0.5 cm^−1^.

An Attension Theta tensiometer was used to measure the contact angle of each prepared membrane using the sessile drop method. Through a very fine capillary, 4 µL of a deionized water droplet was applied to the membrane surface, and the contact angle was determined dynamically using One Attension image analysis software.

The gravimetric approach was used to calculate the porosity (***ε_m_***) of the blend and each composite membrane. The average membrane porosity was concluded as the overall void fraction, estimated as the volume of the pores divided by the total volume of the membrane. Completely dehydrated membrane samples were weighed with a precision balance. Individual membrane samples were then immersed in water and kerosene for 24 h and weighed again. Equation (2) was utilized to determine the porosity (%) of each membrane sample.

(2) $$Porosity\;\left(\%\right)=\frac{W_{w}-W_{d}}{\rho_{w}A\delta }\times 100\;\%,$$

where *W_d_* is the dry sample weight (g), *W_w_* is the wet sample weight (g), *ρ_w_* is the density of pure water and kerosene (g/cm^3^), *δ* is the membrane thickness in the wet state (cm), and *A* is membrane area in the wet state (cm^2^).

The tensile strength and percentage elongation of the PLA/PBS blend and PLA/PBS/MWCNT composite membranes were examined using an Instron 5566 with a load cell of 1 kN at 25 °C. The 6 cm long specimen was placed in the machine with a 50 mm/min crosshead speed. Three tests of each sample were performed to obtain the tensile strength, elastic modulus, and percentage elongation at break.

The morphologies of the PLA/PBS blend and MWCNT/PLA/PBS composite membrane were observed using a JSM-7600F field-emission scanning electron microscope (JEOL, Tokyo, Japan) at an accelerating voltage of 10 kV. The samples were prepared cryogenically by dipping them in liquid nitrogen. Furthermore, the prepared samples were positioned on a metal holder and sputter-coated with gold under vacuum. Finally, the surface and cross-section micrographs were obtained by SEM.

### 2.3. Membrane Performance

**Figure 1:**
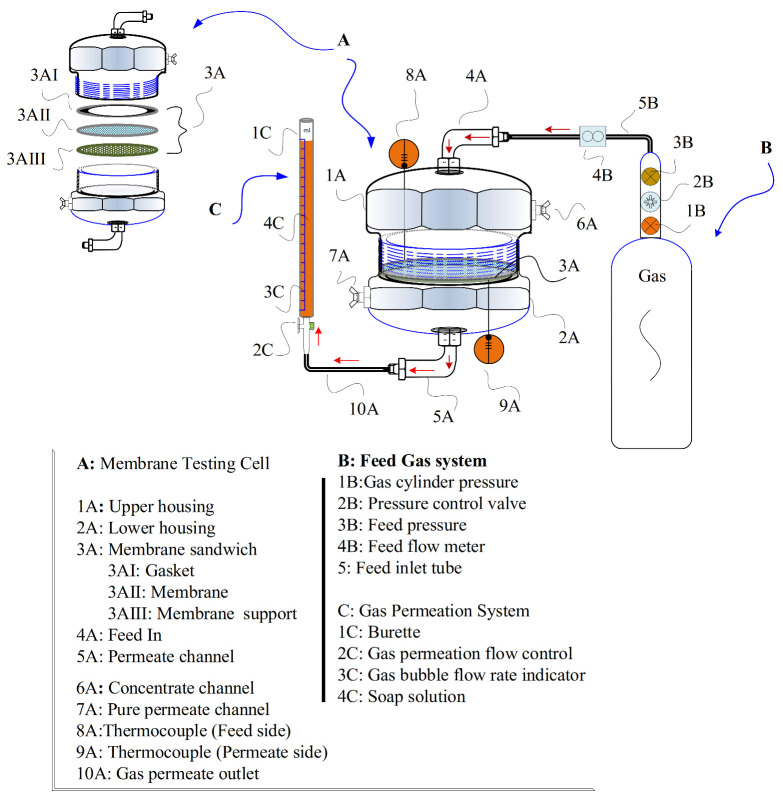
Illustration of gas permeation experiment setup (membrane cell design (permission from [24] US Patent US10935474B1).

Figure 1 shows a comprehensive illustration of the gas permeation experiment setup to evaluate the membrane performance, using a composite plastic cell for the investigation. The membrane cell consisted of detachable separate circular sections attached with the help of a clamp. The feed and retentate gas stream was connected to the upper part of the cell, while the lower part was used as an exhaust for permeated gas streams. A detailed description of the membrane gas separation cell is summarized in Figure 1.

The circular membrane discs provided a 12.5 cm^2^ permeation area. The permeation was performed for Ar, N_2_, CO_2_ and H_2_ at 2 bar, 3 bar, and 4 bar (25 °C). During the experiment, the pressure was maintained at 1 bar on the permeate side, providing a Δ*p* of 1 bar, 2 bar, and 3 bar. Furthermore, a soap bubble flow meter was used to measure the volumetric flow rate of the standard permeate. Equation (3) was utilized to calculate the gas permeance.

(3) $${\left(\frac{P}{l}\right)}_{i}=\frac{Q_{i}}{A\Delta p},$$

where *Qi* is the gas volumetric flow rate (cm^3^ (STP)/s), *A* is the membrane surface area (cm^2^), *l* is the membrane thickness (cm), and Δ*p* is the pressure difference across the membrane (bar). The common unit of permeance is GPU, which is equal to 10^−6^ cm^3^ (STP)/cm^2^·s·cm Hg [19]. Equation (4) was utilized to calculate the selectivity as an ideal separation factor αi/ji at 4 bar.

(4) $$\alpha_{i/j}=\frac{P_{i}}{P_{j}}.$$

## 3. Results and Discussion

**Figure 2:**
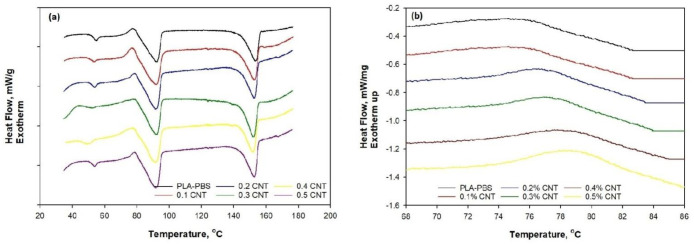
DSC thermogram of MWCNT/PLA/PBS composite: (**a**) heating; (**b**) cooling.

The heating DSC thermograms of PLA/PBS and MWCNT/PLA/PBS are shown in Figure 2a. Two exothermic peaks for PLA and PBS are shown in the thermogram at 97.2 °C and 153.4 °C, corresponding to the melting temperature. The results illustrate that PBS and PLA were immiscible to some extent, possibly due to their melting behavior as a function of the variable crystal size in the blend [12]. The glass transition temperature for PLA is also presented at 54.8 °C.

Figure 2b shows the cooling curve for the crystallization behavior of MWCNT/PLA/PBS. The crystallization exotherm was barely noticeable for PLA as it crystallized slowly, exhibiting a single exothermic peak for the PLA/PBS blend, which corresponded to PBS.

In the DSC cooling cycle for the PLA/PBS blend, we can notice the single exothermic peak for PBS; technically, such peaks occur because of quick crystallization behavior. PBS crystallizes at 79.8 °C, which might also help to crystallize PLA on a minute level. As PBS crystallizes faster, it can act as a nucleation site to crystallize the PLA/PBS blend up to 3.1%. Figure 2a shows the influence of MWCNT on PLA/PBS blend thermal behavior with a heating rate of 2 °C/min. The exotherm simultaneously shows the melting behavior of PLA and PBS crystals at 153.5 °C and 97.2 °C, respectively. The melting temperature increasedby 2 °C when 0.5 wt.% MWCNT was added to the PLA/PBS blend. As the MWCNT content increased in the PLA/PBS blend, the melting temperature also increased.

Additionally, MWCNT could also function as a nucleation site for PBS crystallization, reaching up to 6.1% with 0.5 wt.% MWCNT, as shown in Figure 2b. The crystallization aided by the presence of MWCNT might have occurred in the PBS matrix and in the vicinity of interfaces around PLA/PBS segments. Figure 2b also demonstrates that the addition of MWCNT increased the peak magnitude, illustrating that PBS in the PLA/PBS blend crystallized at higher temperatures. Nevertheless, the Tgof the blend composite membrane decreasedby 2 °C to 3 °C, possibly due to the MWCNT concentration used in this study. The decreasing trend of Tg and increasing trend of Tc revealed the influence of MWCNT on the composite membrane. However, the difference was not significant for low amounts of MWCNT.

Figure 2b also demonstrates that the addition of MWCNT increased the peak magnitude, illustrating that PBS in the PLA/PBS blend crystallized at higher temperatures. The increase in the peak magnitude occurred because MWCNT helped to create additional nucleation sites, which magnified the crystallization in MWCNT/PLA/PBS [25]. The thermal properties of the PLA/PBS blend membrane and MWCNT/PLA/PBS composite membranesare summarized in Table 1.

**Figure 3:**
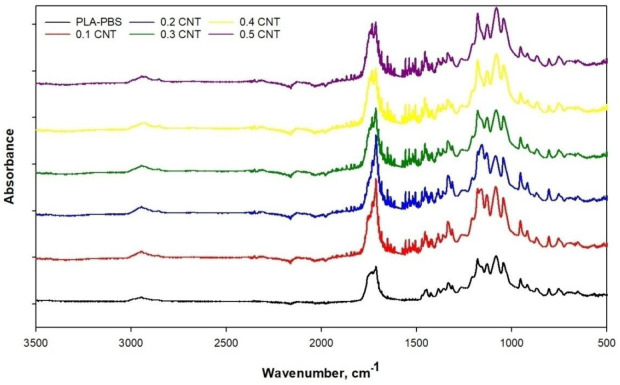
FTIR of PLA/PBS blend membrane and MWCNT/PLA/PBS composite membranes.

Figure 3 shows the FTIR results of the PLA/PBS blend membrane and MWCNT/PLA/PBS composite membranes. Technically speaking, the FTIR results reflect the structural changes occurring in PLA/PB and MWCNT due to the physisorption reactions between the three components used in the membrane.

The characteristic peaks of the PLA/PBS were as follows: 2945—symmetric stretching of CH_3_, 1737—C=O stretching to PLA and PBS, C–OH bending of PBS corresponding to CH_2_ twisting in PBS, 1386—symmetric bending in PLA and PBS for CH_3_, 1447—asymmetric bending of CH_3_, 1184—asymmetric C−O−C and asymmetric rocking CH_3_, 1121—symmetric rocking of CH_3_, 1082—symmetric stretching of C−O−C, 1044—stretching of C−CH_3_, and 870—stretching of C−COO, also attributed to the amorphous region of the PLA phase. Moreover, the carbonyl peak at 1737 cm^−1^ in the PLA/PBS blend was broader, representing that PLA and PBS are miscible, obtaining a relatively homogeneous and stable blend due to the adhesive tendency of PLA. The miscibility was present because of interactions between C–O or C=O and the hydroxyl group –OH found at the end of PLA and PBS chains. Generally speaking, hydroxyl is present in both polymer structures; thus, the appearance of the –OH group suggests homogeneous mixing between the two polymers. A similar trend was also observed when we mixed MWCNT in the blend solution.

The multiple appearances of repeated –OH was potentially revealed due to the hygroscopic nature of MWCNT. Furthermore, the stable blend dope revealed an efficient dispersion of MWCNT in chloroform and the tendency of PLA adhesion properties in a nonpolar solvent. The addition of 0.5 wt.% MWCNT to the PLA/PBS blend shifted the peak bands from 2996 cm^−1^ to 2990 cm^−1^ and 1748 cm^−1^ to 1743 cm^−1^ because of structural changes due to the higher number of functional groups present in the composite membranes. Furthermore, the inclusion of MWCNT in PLA/PBS introduced characteristic peaks at 1066 cm^−1^ and 1176 cm^−1^ for C–O, 1600 and 1450 cm^−1^ for the aromatic ring, and 1750 cm^−1^ for C=O. The clear peak absorption of the aromatic rings probably revealed an interlinking between PLA and PBS. Simultaneously, the equal and uniform distribution of the MWCNT molecule showed a significant agreement with the composite membrane. Peaks were formed at 1750 and 1066 cm^−1^ for MWCNTs because of C=O stretching vibration and C–O in the carboxyl group. An apparent absorption peak can be noted at 1750 cm^−1^ related to C=O stretching, shows the formation of robust van der Waals force due to the significant lattice and adhesion property of PLA, which seemingly led to efficient affinity between PLA/PBS and MWCNT.

**Figure 4:**
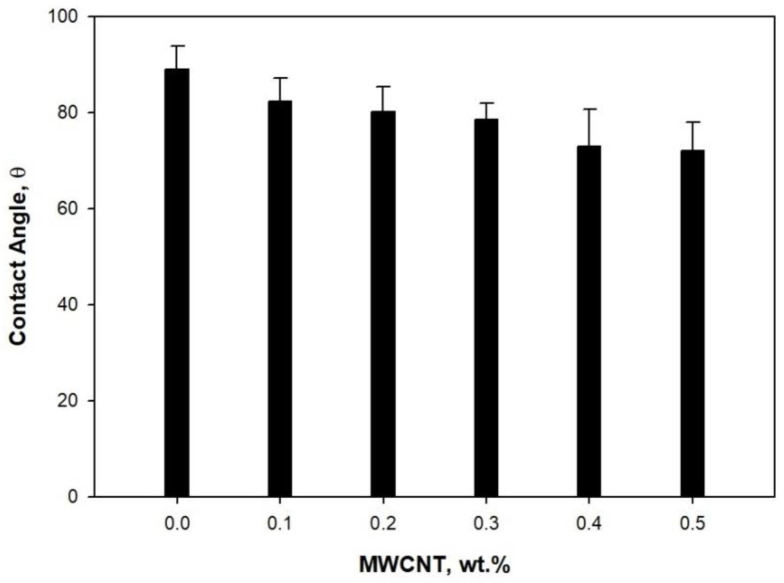
Contact angle of MWCNT/PLA/PBS composite.

The contact angle for the PLA/PBS blend and MWCNT/PLA/PBS composite membranes is shown in Figure 4. The contact angle for neat PLA/PBS was about 89.0°, representing a hydrophobic membrane. The hydrophobicity of PLA/PBS was probably due to the higher crystalline affinity of PLA, as supported by the results of DSC. The addition of MWCNT to the PLA/PBS blend altered the physical characteristics of the composite membrane, making it hydrophilic. The membrane with 0.5 wt.% MWCNT had a contact angle of approximately 71.98°.

Moreover, this 20% reduction in contact angle indicated the hygroscopic nature of MWCNT (0.5%) in the PLA/PBS composite membrane. These results revealed a significant presence of MWCNT in the composite membrane. The FTIR results support the homogeneous dispersion of MWCNT in the PLA/PBS composite membranes. Furthermore, it also predicted that the carboxylic groups present in MWCNT increased the hydrophilicity of the membranes.

**Figure 5:**
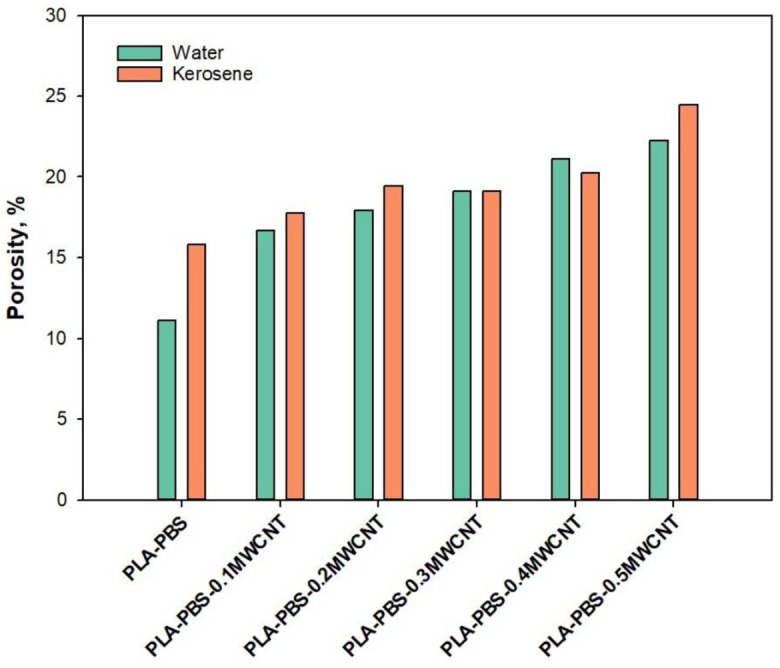
Porosity of MWCNT/PLA/PBS composite.

Figure 5 shows the effect of adding MWCNT to PLA/PBS on the porosity of the membrane. The neat PLA/PBS membrane had a porosity of 11.1% for water and 15.82% for kerosene. The porosity increased from 11.1% to 22.1% for water and from 15.82% to 24.43% for kerosene with the addition of 0.5 wt.% MWCNT. The hydrophilicity was enhanced because MWCNT allowed more nonsolvent such as ethanolto enter the casting solution, thereby increasing the solvent exchange inside the membrane [26]. The number of empty spaces might have increased in the membrane structure because of higher solvent and nonsolvent exchange during the phase inversion process. The empty spaces also improvethe porosity of the membrane. The addition of MWCNT to the PLA/PBS membrane possibly increased the number of useful empty spaces and magnified the porosity [27,28,29]. During the gas separation process, this can aid in efficient gas diffusion.

The life span of the membrane can be increased by the inclusion of MWCNT as it adds to the overall strength and prevents breakage [30]. The flexibility of MWCNT allows the membrane to bend instead of breaking when force is applied, as well as assists in retaining the membrane’s original shape when the applied force is removed. The flexible nature of MWCNT improved the tensile strength of the polymer matrix. Table 2 represents the mechanical properties of the PLA/PBS blend and MWCNT/PLA/PBS composite membranes.

There was an increase in the tensile strength of the PLA/PBS membrane when the amount of MWCNT was increased. The neat PLA/PBS membrane had a tensile strength of 9.3 ± 0.41 MPa. The increase in tensile strength (30%) was most noticeable for 0.5 wt.% MWCNT.

Furthermore, 0.5 wt.% MWCNT increased the elastic modulus of the PLA/PBS membraneby 13% and reduced its elongation at break by 10%. This decrease in percentage elongation at break might have occurred due to the brittleness of PLA and the existence of MWCNT resisting the molecular chain mobility of PLA/PBS. The addition of MWCNT to the PLA/PBS membrane improved tensile strength and elastic modulus, in agreement with the literature [31,32]. The improved tensile strength of the MWCNT/PLA/PBS membrane possibly resulted from the increased interaction between MWCNT and the PLA/PBS matrix.

**Figure 6:**
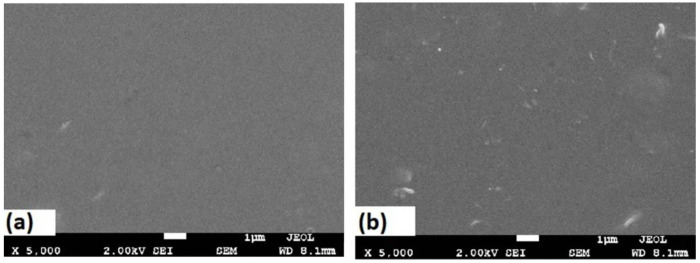
SEM micrographs of surface images: (**a**) PLA/PBS blend; (**b**) 0.5 MWCNT/PLA/PBS composite membrane.

**Figure 7:**
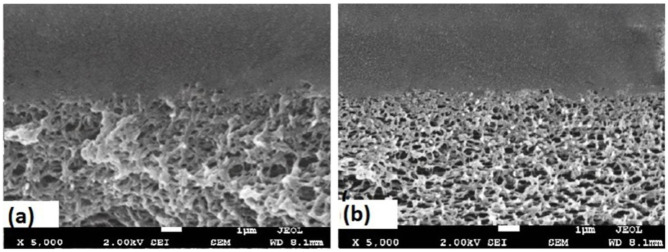
SEM micrographs of cross-sectional images: (**a**) PLA/PBS blend; (**b**) 0.5 MWCNT/PLA/PBS composite membrane.

The best membrane was selected for SEM analysis; Figure 6a,b illustrate the SEM micrographs for the surfaces of the PLA/PBS blend and MWCNT/PLA/PBS composite membrane. In addition, the cross-section for the neat PLA/PBS blend and MWCNT/PLA/PBS composite membrane is shown in Figure 7a,b. The morphological results of the PLA/PBS blend and MWCNT/PLA/PBS composite membrane showed dense asymmetry in the structure; however, the cross-section (Figure 7a,b) revealed noticeable macropores in the membrane. Figure 6a shows a neat PLA/PBS membrane with a defect-free moderately smooth surface, whereas Figure 6b demonstrates a uniform dispersion of 0.5 wt.% MWCNT in the PLA/PBS matrix. However, the membrane surface revealed more roughness as compared to the PLA/PBS blend, which could possible enhance gas diffusion. Moreover, Figure 7a shows the absence of lattice pores in the neat PLA/PBS membrane. Agglomeration was not observed because of the strong interaction between the PLA/PBS matrix and MWCNTs.

Figure 7b shows that the quantity and density of tiny pores increased with the inclusion of MWCNT, as compared to the neat PLA/PBS membrane shown in Figure 7a. The improvement in porosity might have occurred due to the increase in viscosity of membrane solution with the addition of MWCNT [33,34,35]. The solvent–nonsolvent exchange rate was decreased with the increase in viscosity during phase separation. Furthermore, Figure 7b demonstrates the homogeneous dispersion of voids because of the intense interaction between MWCNTs and PLA/PBS. Moreover, the van der Waals forces and π–π stacking between PLA/PBS molecular chains and aromatic rings enhanced the interfacial adhesion, thereby improving the distribution of MWCNTs and increasing the solution viscosity. The addition of MWCNT to the PLA/PBS membrane produced fewer interface voids and prevented agglomeration in the membrane, thus allowing better gas permeance.

**Figure 8:**
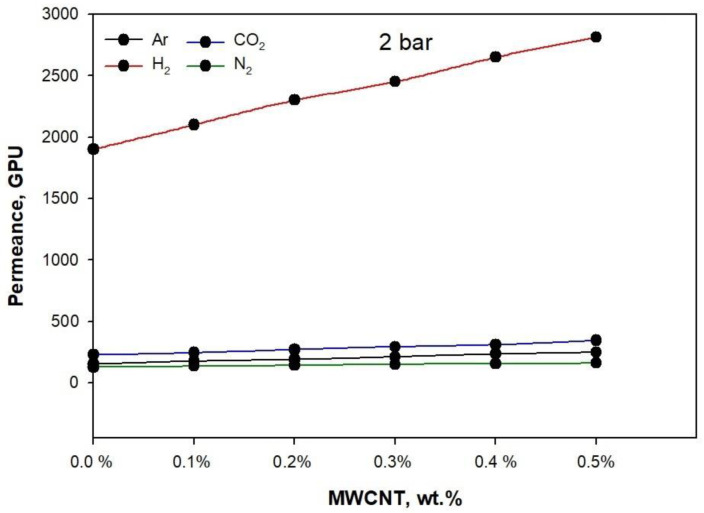
Gas permeance performance at 2 bar of PLA/PBS blend and MWCNT/PLA/PBS composite membrane.

**Figure 9:**
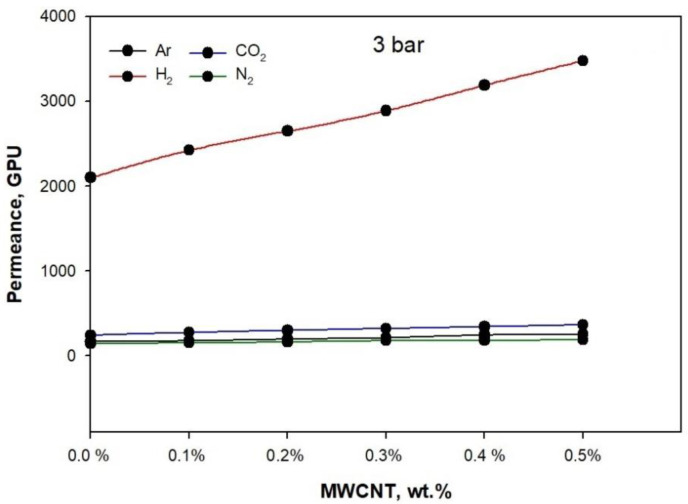
Gas permeance performance at 3 bar of PLA/PBS blend and MWCNT/PLA/PBS composite membrane.

**Figure 10:**
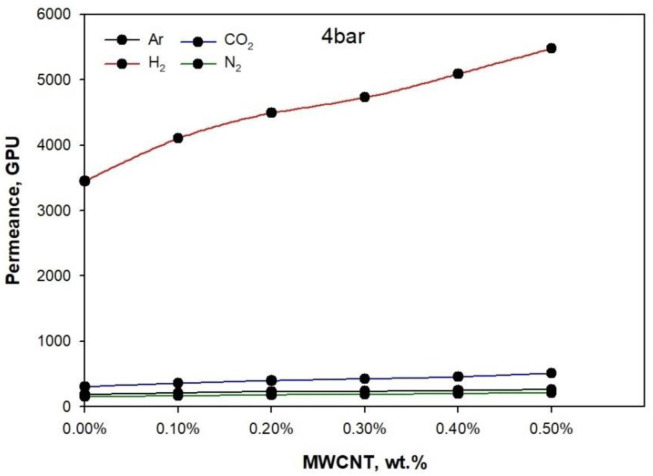
Gas permeance performance at 4 bar of PLA/PBS blend and MWCNT/PLA/PBS composite membrane.

Figure 8 (2 bar), Figure 9 (3 bar), and Figure 10 (4 bar) illustrate the permeance of Argon (Ar), Hydrogen (H_2_), Nitrogen (N_2_), and carbon dioxide (CO_2_), calculated by analyzing the thickness of the membrane. At 25 °C, the permeation measurements were performed using pure gases (H_2_, Ar, N_2_, and CO_2_) at feed pressures of 2 bar, 3 bar, and 4 bar.

Figure 8, Figure 9 and Figure 10 show the effect of rising pressure on permeance from 2 bar to 4 bar for the neat PLA/PBS blend. The permeance increased for H_2_ from 1900.6 GPU to 4090.5, for CO_2_ from 227.71 GPU to 282 GPU, for Ar from 151.12 GPU to 187.3 GPU, and for N_2_ from 127.21 to 155.2 GPU, i.e., H_2_ > CO_2_ > Ar > N_2_. The rise in pressure from 2 bar to 4 bar increased Δp (1, 2, and 3 bar), which acted as a strong driving force for the adequate permeation of gases. The PLA/PBS membrane characteristics of void volume, chain mobility, gas solubility, and gas molecular sizefacilitated gas permeation [36,37]. The increase in MWCNT from 0.1 to 0.5 wt.% enhanced the gas permeance. It can be observed that the addition of 0.5 wt.% MWCNT at 4 bar increased the permeance for H_2_ to 5478.29 GPU, for CO_2_ to 510.2 GPU, for Ar to 262.8 GPU, and for N_2_ to 209.09 GPU when compared to thepermeance at 2 bar. The high permeance of H_2_ possibly resulted from the increased narrow free volume in pores produced as a function of the improved interaction between MWCNT and PLA/PBS. Moreover, in the MWCNT/PLA/PBS membrane, the increase in free volume improved gas permeance compared to the neat PLA/PBS membrane. Furthermore, Figure 2 shows that the improved percentage crystallinity in the PLA/PBS/MWCNT membrane also facilitated gas permeance. The results show that an increase in percentage crystallinity also improved gas permeance. The gas permeance for the 0.5%MWCNT/PLA/PBS membrane with 6.5% crystallinity was higher than that for the neat PLA/PBS membrane with 3.1% crystallinity.

**Figure 11:**
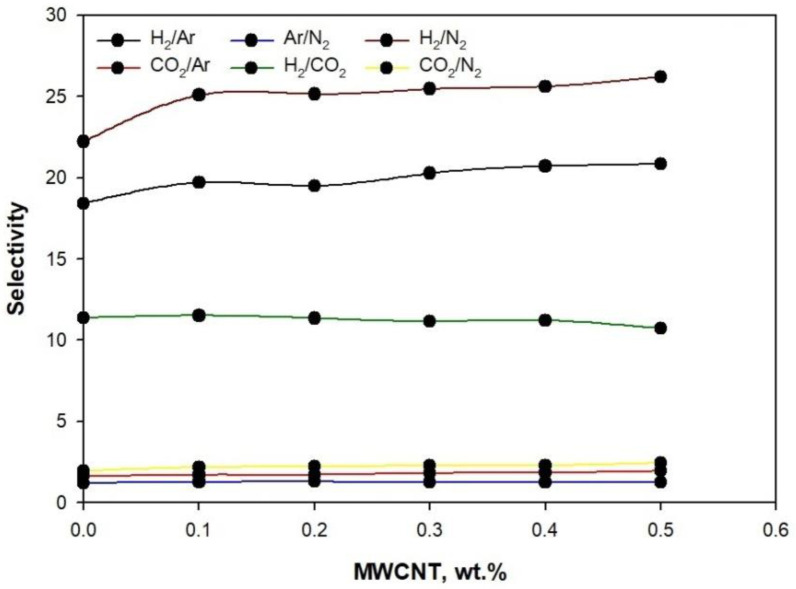
Selectivity of gases at 4 bar.

Figure 11 illustrates the ideal selectivity measured at 4 bar for H_2_/Ar, H_2_/N_2_, H_2_/CO_2_, CO_2_/N_2_, CO_2_/N_2_, and Ar/N_2_ for neat PLA/PBS and MWCNTs/PLA/PBS membranes.The addition of 0.5 wt.% MWCNT to PLA/PBS increased the selectivity for H_2_/N_2_ from 22.2 to 26.21 compared to neat PLA/PBS, which was higher than that observed for other gases. The increase in selectivity for H_2_/N_2_ may have occurred due to the improved interfacial interaction between the PLA/PBS chain and MWCNT. In contrast, the selectivity for Ar/N_2_ in MWCNT/PLA/PBS was lower by ~1.25. The functional groups present on MWCNT influenced the gas selectivity of MWCNT/PLA/PBS membranes. The addition of MWCNT from 0.1 wt.% to 0.5 wt.% in PLA/PBS did not result in a sufficient improvement in selectivity for CO_2_/N_2_, CO_2_/Ar, and Ar/N_2_. The higher selectivity of H_2_/N_2_ (26.21) is evidence of molecular separation. Hydrogen, with its smaller kinetic diameter (0.28 nm), permeated more quickly than nitrogen (0.36 nm) and effectively entered into the network of pores. Compared to H_2_, kinetic constraints hindered the entrance of N_2_ molecules into the pore structure, thus providing a high selectivity for H_2_/N_2_.

Moreover, the propagation of N_2_ through the pores may have been interrupted compared to H_2_ because of a higher level of adsorption happening due to the close proximity of pore walls. The selectivity for H_2_/CO_2_ was lower, ~10.8, when compared to H_2_/N_2_. The results greatly depend on adsorption potential, which influences the diffusion rate of molecules through pores. Furthermore, H_2_ performed better than CO_2_ and Arin terms of separation through the MWCNT/PLA/PBS membrane. This efficient H_2_ separation performance could be attributed to suitable micropore diffusion when compared to adsorption ability. The kinetic diameter of H_2_ is 0.28 nm, which assists in its relevant passage through pores, therebyfacilitating gas permeance, compared to CO_2_ (0.33 nm) and argon (0.34) nm.

## 4. Conclusions

The phase inversion method was used to develop MWCNT-reinforced PLA/PBS membranes. MWCNT in the PLA/PBS matrix led to improvements by forming additional nucleation sites, physically interlinking aromatic groups, and introducing van der Waals forces, as recognized by DSC and FTIR. The inclusion of 0.5 wt.% MWCNT in PLA/PBS increased the porosity, which was attributed to enhanced hydrophilicity, and it revealed significant gas dissolution and diffusion selectivity. The SEM micrographs showed the adequate dispersion of MWCNT in the PLA/PBS matrix. The improved interfacial adhesion and absence of voids led to the MWCNT/PLA/PBS membrane being capable of elevated permeance and increased selectivity for H_2_/N_2_. The gas permeance acquired at 25 °C and 4 bar for PLA/PBS reinforced with MWCNT was found to be highest in hydrogen, followed by carbon dioxide, argon, and nitrogen. The increase in Δp improved permeance through the membrane. The improvement in selectivity for H_2_/N_2_ could be attributed to enhanced interactions among MWCNT, PLA, and PBS chains.

## Figures and Tables

**Table 1:** Thermal properties of MWCNT/PLA/PBS membrane.

Materials	Tg, °C	Tc, °C	Tm, °C		ΔHm, J/g		X_c_, %
			PBS	PLA	PLA	PBS	
PLA–PBS PLA–PBS–0.1 CNT PLA–PBS–0.2 CNT PLA–PBS–0.3 CNT PLA–PBS–0,4 CNT PLA–PBS–0.5CNT	54.8 53.5 53.2 52.7 52.6 52.0	79.9 80.1 80.6 81.2 81.8 82.2	94.8 95.1 95.7 96.1 96.7 97.2	150.3 151.5 152.4 152.6 152.9 153.5	8.03 8.60 8.83 7.23 6.70 8.55	8.85 9.53 9.99 10.80 9.62 7.54	3.1 3.8 4.1 5.4 6.1 6.5

**Table 2:** Mechanical properties of PLA/PBS blend and MWCNT/PLA/PBS composite membranes.

Materials	Tensile Strength, MPa	Elongation at Break, %	Young Modulus, MPa
PLA–PBS PLA–PBS–0.1 CNT PLA–PBS–0.2 CNT PLA–PBS–0.3 CNT PLA–PBS–0,4 CNT PLA–PBS–0.5CNT	9.3 ± 0.41 9.9 ± 0.35 10.7 ± 0.31 11.5 ± 0.29 12.7 ± 0.31 13.4 ± 0.25	65.53 ± 0.04 64.37 ± 0.06 63.19 ± 0.05 61.78 ± 0.04 60.56 ± 0.03 59.48 ± 0.05	456 ± 4.8 468 ± 4.7 481 ± 4.1 497 ± 5.6 511 ± 4.9 519 ± 5.8

## Data Availability

Not applicable.
